# ATP-Citrate Lyase Supports Cardiac Function and NAD^+^/NADH Balance and Is Depressed in Human Failing Myocardium

**DOI:** 10.1016/j.jacbts.2025.04.015

**Published:** 2025-06-10

**Authors:** Mariam Meddeb, Navid Koleini, Mohammad Keykhaei, Ting Liu, Marcus Rhodehamel, Lorena Mandarano, Farnaz Farshidfar, Liang Zhao, Seoyoung Kwon, Gizem Keceli, Ismayil Ahmet, Nazareno Paolocci, Virginia Hahn, Kavita Sharma, Erika L. Pearce, David A. Kass

**Affiliations:** a Division of Cardiology, Department of Medicine, Johns Hopkins University School of Medicine, Baltimore, Maryland, USA; b Complete Omics Inc, Halethorpe, Baltimore, Maryland, USA; c Laboratory of Cardiovascular Sciences, National Institute of Aging, Baltimore, Maryland, USA; d Department of Oncology, Department of Biochemistry and Molecular Biology, Johns Hopkins University School of Medicine, Baltimore, Maryland, USA; e Department of Pharmacology and Molecular Sciences, Johns Hopkins University, Baltimore, Maryland, USA

**Keywords:** heart disease, metabolism, myocardium, redox, reductive stress, TCA cycle

## Abstract

ATP-citrate lyase (ACLY) regulates lipogenesis and cell proliferation, and forms a cytosolic TCA-bypass circuit impacting NADH. We show that acute and chronic ACLY inhibition in cardiomyocytes depresses the NAD^+^/NADH ratio by increasing mitochondrial NADH. Acute suppression causes dose-dependent cytotoxicity, but at low doses augments aerobic respiration without impeding myocyte function. ACLY is reduced in human failing myocardium, and mice with myocardial or myocyte ACLY knockdown display mildly depressed function, particularly after pressure-overload, and exertional limitations. NAD+ enhancement ameliorates dysfunction/toxicity from ACLY inhibition. These results reveal that ACLY intrinsically regulates cardiac NAD^+^/NADH balance and respiration, which can affect rest and reserve heart function.

## Highlights

• In cardiomyocytes, the enzyme ACLY constitutively blunts generation of mitochondrial NADH used for oxidative respiration.
• ACLY inhibition by gene suppression or small molecule inhibitors increases mitochondrial NADH lowering NAD+/NADH ratio, acutely stimulating oxidative respiration while also inducing dose-dependent cytotoxicity.
• Reducing cardiomyocyte ACLY in vivo causes mild systolic dysfunction that is amplified after pressure-load stress.
• Myocyte cytotoxicity and in vivo chamber dysfunction are rescued by augmenting NAD+ to restore the NAD+/NADH balance.
• Human failing myocardium (both reduced and preserved ejection fraction) exhibits reduced ACLY expression, potentially contributing to reduced NAD+/NADH levels and rest and reserve function.

## Visual Abstract

**Figure:**
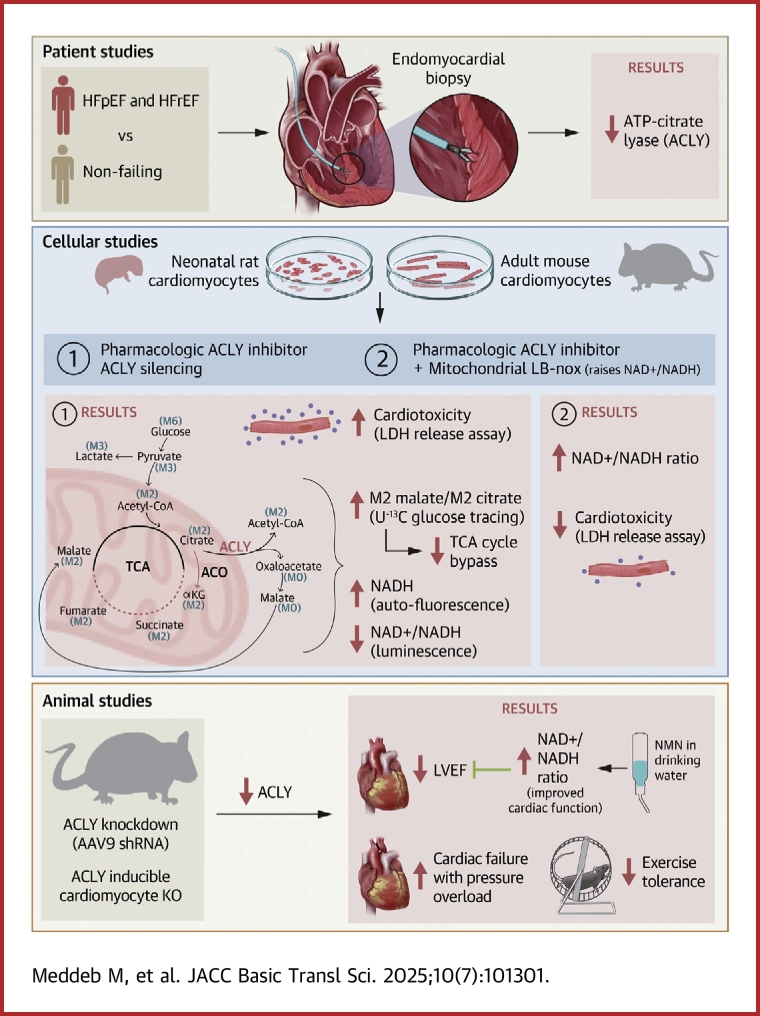

ATP citrate lyase (ACLY) is a highly conserved cytosolic enzyme catalyzing the reaction whereby citrate exported from mitochondria is combined with coenzyme A (CoA) to form oxaloacetate (OA) and acetyl-CoA.^1^ The acetyl-CoA can be used for fatty acid and cholesterol synthesis and provides acetyl groups for protein acetylation.^2^ In liver and adipocytes, ACLY plays an important role linking glucose metabolism to lipogenesis and membrane formation and is associated with hyperlipidemia.^3^ Bempedoic acid, a pro-drug ACLY inhibitor requiring activation by a hepatocyte-specific enzyme, is clinically approved for hyperlipidemia.^4^ ACLY also fuels cancer^5^ and vascular smooth muscle proliferation,^6^ and its in vivo inhibition is being pursued as a potential therapy for pulmonary hypertension and coronary vascular disease,^6^ obesity and ectopic lipid accumulation in diabetic kidney disease,^7^ and cancer.^8^ ACLY plays additional epigenetic roles via histone acetylation, eg, regulating myofibroblast differentiation and persistence.^9^

ACLY also integrates the canonical tricarboxylic acid cycle (TCA) with a cytosolic bypass circuit to modulate metabolism. OA generated by ACLY from TCA-derived citrate is further reduced to malate in the cytosol, which can then re-enter mitochondria to join the TCA cycle.^10^ This bypasses 2 nicotinamide adenine dinucleotide (NADH)-generating steps in the TCA, with another NADH being consumed in the cytosol. This ACLY-dependent cycle is prominent in proliferating cells such as cancer, wherein ACLY depletes cytosolic citrate and NADH, activates glycolysis,^11^ and provides cytosolic acetyl-CoA for protein acetylation and membrane synthesis.^5^

Cardiomyocytes also express ACLY, but its impact on metabolic and functional properties remains largely unknown. Because the heart requires substantial NADH to fuel oxidative respiration, and this is mostly provided by the TCA cycle, a potential bypass that would diminish NADH formation could position ACLY as a metabolic regulator. This has potential translational relevance because prior myocardial transcriptomic analysis of human heart failure with reduced ejection fraction (HFrEF) or heart failure with preserved ejection fraction (HFpEF) found *ACLY* expression significantly reduced.^12^ Here, we tested the role of ACLY in myocytes and intact hearts using pharmacological and/or genetically induced ACLY suppression. We find that ACLY suppression lowers the NAD^+^/NADH ratio, acutely increases mitochondrial respiration, and its chronic reduction results in modest cardiac dysfunction. The latter is exacerbated by cardiac stress and rescued by NAD+ augmentation in vivo.

## Methods

### Ethical approvals

Studies of human myocardial tissue were performed under an Institutional Review Board–approved nonclinical research exempt protocol. Mouse studies were performed under a protocol approved by the Johns Hopkins School of Medicine Committee on Animal Care, in compliance with all standing ethical regulations for the use of animals in research.

### Pharmacological ACLY inhibition

ACLY was pharmacologically inhibited primarily using BMS-303141 (Tocris #4609) dissolved in DMSO at concentrations identified in the experiments. Several other inhibitors served as comparators (SB-204990 [MedChem Express #HY-16450], NDI-091143 [Selleckchem #S8878], and 10,11-dehydrocurvularin [MedChem Express #HY-N6679A]). When used, the term ACLYi refers to BMS-303141.

### Cardiomyocyte isolation

Adult mouse cardiomyocytes (AMCM) were isolated using a Langendorff free method.^13^ Myocytes were plated in M199 media containing 10% FBS for 1 hour, then media changed to serum-free M199 supplemented with Insulin Transferrin Selenium (ThermoFisher Scientific #51500056), 1:100 Chemically Defined Lipid Concentrate (Gibco #11905031) and 2,3-butandione monoxime (Sigma-Aldrich #B0753). Neonatal rat ventricular cardiomyocytes (NRCM) were isolated as described.^14^

### Cytotoxicity and reactive oxygen species assays

Myocyte cytotoxicity was assessed by lactate dehydrogenase (LDH) release into the media using the LDH-Glo assay (Promega) per manufacturer’s instructions. Reactive oxygen species (ROS) were assayed using electron paramagnetic resonance, the fluorescent mitochondrial ROS sensor MitoSOX, and cytosolic H_2_O_2_ sensor cytoORP1; details in the Supplemental Methods. In some studies, ROS suppression by N-acetyl-L-cysteine (200 μmol/L, Sigma Aldrich #A9165) or Dithiothreitol (0.5 mmol/L DTT, ThermoFisher #R0861) added to the NRCM or adult myocyte culture medium 4 hours before the cells were exposed to ACLYi (25 μmol/L for NRCM, 1 μmol/L for AMCM) or vehicle for an additional hour.

### Metabolic assays

Metabolic tracing was performed in freshly isolated AMCM in 6 well plates in glucose free-M199 medium (ThermoFisher) supplemented with 5.5 mmol/L of uniformly labeled U^13^C-glucose, 1:100 Chemically Defined Lipid Concentrate (CD lipids; Gibco #11905031), Insulin-Transferrin-Selenium (Gibco#51500056), and 2,3-butandione monoxime (Sigma-Aldrich #B0753). Myocardial NAD^+^/NADH was determined by NAD/NADH-Glo^TM^ (Promega #G9072) per manufacturer’s instructions in myocyte and mitochondrial lysates (Supplemental Methods).

### Adult myocyte functional studies

Freshly isolated AMCMs were exposed to 1 μmol/L ACLYi or vehicle control for 1 hour, and myocyte function (IonOptix) and whole cell calcium transients (Fura-2 AM) were determined. Cells were perfused with phenol red free M199 media at a continuous flow rate of 1 mL/min, and electrically stimulated at 1 Hz. Data were obtained from ∼15 randomly selected myocytes per field, repeated in at least 3 separate aliquots from each of 4 separate hearts.

### Mitochondrial assays

Methods for fluorescent measurement of mitochondrial membrane potential, cellular respiration in NRCMs using aXF96 Seahorse Analyzer, mitochondrial isolation and respiration assays,^15^ and real-time mitochondrial NADH assay^16^ are provided in Supplemental Methods.

### RNA extraction, semiquantitative real time RT-PCR, and protein immunoblot analysis

Details in the Supplemental Methods, Supplemental Tables 2 and 3.

### In vivo mouse models

C57BL/6J mice (Jackson Labs, #000664) were housed in the JHU Animal Care facilities at 25 ^o^C in 45% humidity under 12-hour light/dark cycles. Unless specified, mice were fed standard chow and had access to tap water ad libitum.

#### Tamoxifen-inducible cardiomyocyte-specific ACLY knock-down

Mice (c*Acly*^*+/+*^) were generated by crossing floxed *Acly* mice (Jackson Labs, MMRC stock #43555) to αMHC-MerCreMer mice (Jackson Labs, #005657). The 8- to 10-week-old C57BL6/J mice were fed with an 80 mg/kg tamoxifen diet (Inotiv #TD.130858) for 8 days and then returned to a regular diet. Age-matched αMHC-MerCreMer mice without floxed *Acly* served as controls. Echocardiography was performed 8 weeks after completion of the tamoxifen protocol.

#### AAV9 Acly-short hairpin (silencing) RNA model

Cardiac ACLY knockdown was achieved by retroorbital injection of AAV9 expressing *Acly-* short hairpin (silencing) RNA (shRNA) or scrambled shRNA (Vector Biolabs). C57BL6/J mice aged 10 ± 2 weeks were provided 2 × 10^11^ genome copies of one or the other AAV9, and maintained in standard housing and feeding conditions for 8 to 10 weeks. In one subgroup, 4 weeks after AAV9 injection, mice were randomized to receive 200 mmol/L β-nicotinamide mononucleotide (NMN, Chem-Impex #33732) or vehicle added to their drinking water. Echocardiography was performed at the 8- to 10-week timepoint post-AAV9 inoculation in all animals (see Supplemental Methods).

### Statistical analyses

Continuous data are presented using the mean ± SD or median with 25th and 75th percentiles (Q1-Q3), and categorical data as count (percentage). The normality of continuous data was determined using the Kolmogorov-Smirnov test. If normally distributed, 2 groups were compared using Student’s *t*-test and comparisons among multiple groups using 1- or 2-way analysis of variance (ANOVA) or Welch’s ANOVA (significant difference in variance between groups as determined by the Brown-Forsythe test). Longitudinal comparisons used 2-way repeated measures ANOVA. Post-hoc tests for multiple pairwise comparisons employed the methods of Tukey, Sidak, or Dunnett. Non-normally distributed data used the Mann-Whitney *U* (2 groups) or Kruskal Wallis (more than 2 groups). Categorical data were compared using the Fisher exact test.

Statistical analysis were performed with GraphPad Prism version 10.0 (GraphPad Software), and a *P* value < 0.05 was considered statistically significant.

## Results

### ACLY protein expression is depressed in human heart failure

**Figure 1:**
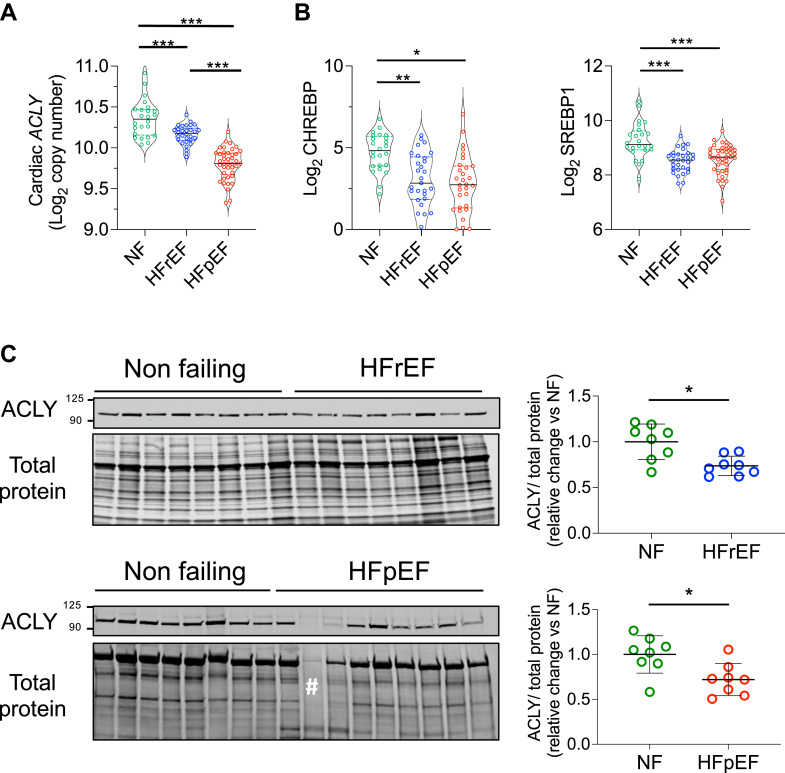
ACLY is Depressed in Human Heart Failure (A) Human myocardial gene expression of ATP citrate lyase (*ACLY*) in nonfailing control subjects, heart failure with preserved ejection fraction (HFpEF), and heart failure with reduced ejection fraction (HFrEF). (B) Gene expression of *CHREBP* and sterol *SREBP1* in the same samples. Normalized read counts are presented on a log_2_ scale. Individual values, median, 25th and 75th percentiles are shown. ANOVA, Tukey’s multiple comparison test; n = 24 nonfailing (NF), 30 HFrEF, and 41 HFpEF patients. (C) Immunoblot of ACLY protein in human HFrEF vs control (top) and HFpEF vs control (bottom) myocardium. Summary densitometry (normalized to total protein). Data shown are mean ± SD, Mann-Whitney *U* test, n = 7-8/group. ∗*P <* 0.05, ∗∗*P <* 0.01, and ∗∗∗*P <* 0.001. #Sample excluded because of very low protein loading.

Figure 1A displays human myocardial *ACLY* gene expression using data from our prior transcriptomic study of HFrEF and HFpEF.^12^ Clinical features of the patient population are provided in Supplemental Table 1. Figure 1B shows results for the primary transcriptional regulators of *ACLY*: carbohydrate response element binding protein (*CHREBP*)^17^ and sterol regulatory element-binding protein-1 (*SREBP1*).^18^^,^^19^ Expression of all 3 genes was significantly below control subjects in both forms of HF, with *ACLY* being the lowest in HFpEF. We further assessed myocardial ACLY protein expression, finding it ∼30% lower in both heart failure groups (Figure 1C).

### Cardiomyocyte ACLY integrates with the TCA cycle

**Figure 2:**
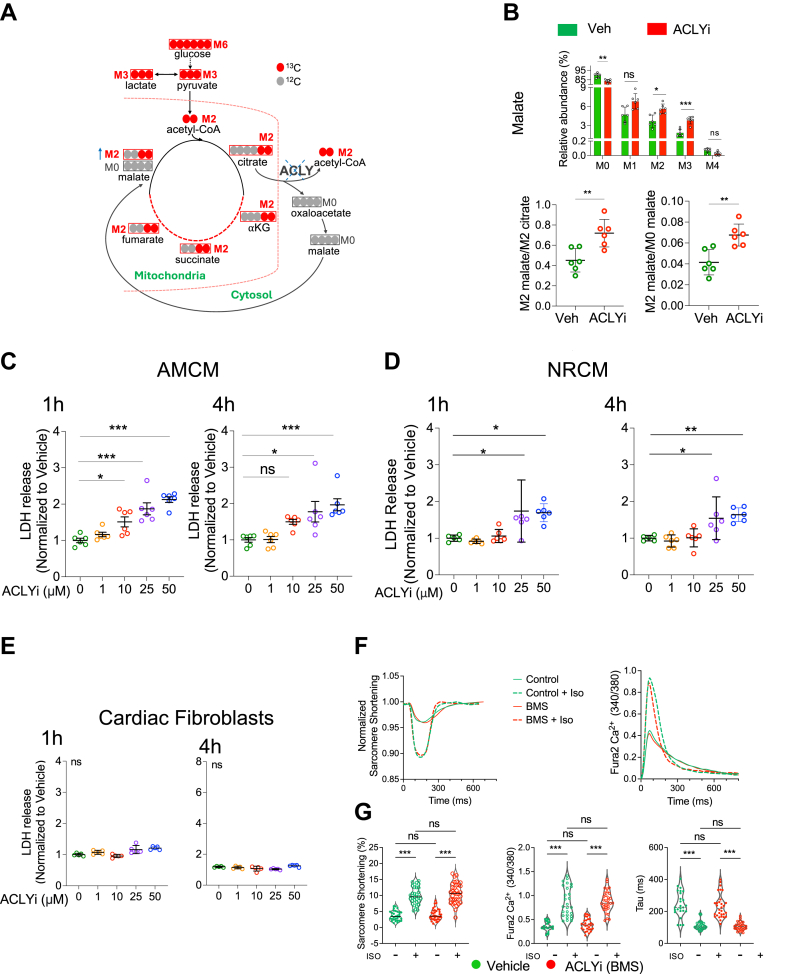
The TCA Cycle Intersects ACLY via Citrate-to-Malate Bypass in Cardiomyocytes (A) Schematic of the catabolism of uniformly-labeled ^13^C-glucose (U-C13 glucose). M+6 U-C13 glucose ultimately generates 2 M+2 acetylCoA, yielding M+2 TCA cycle intermediates (citrate, KG, succinate, fumarate, and malate) if processed through aconitase. If processed by ACLY, the 2 labeled carbons are removed, resulting in nonlabeled M0 malate. (B) Isotopogram of labeled C13 in malate (top); 2-way ANOVA with Sidak multiple comparison test, and ratio of M2 malate/M2 citrate and M2 malate/M0 malate (bottom). Unpaired Student's *t*-test. Data shown are mean ± SD. n = 6/group. (C-E) Change in lactate dehydrogenase (LDH) release normalized to vehicle control from AMCM (C), NRCM (D), and cardiac fibroblasts (E), each exposed to 1-50 μmol/L ACLYi for 1 or 4 hours. n = 6 biological replicates/group, ANOVA, Dunnett’s multiple comparisons test. (F) Sarcomere shortening and calcium transient waveforms from example AMCM after 1 hour exposure to 1 μmol/L BMS or vehicle, with or without isoproterenol (Iso) stimulation. (G) Summary data for sarcomere shortening, peak calcium amplitude, and monoexponential calcium decay time constant (Tau) from the same study. Analysis with Welch’s ANOVA and Dunnett’s multiple comparison’s test. Data shown are individual values, median, 25th and 75th percentiles; n = 31-35 cardiomyocytes/group. ∗*P <* 0.05, ∗∗*P <* 0.01, and ∗∗∗*P <* 0.001.

To determine whether cardiomyocytes engage an ACLY-dependent TCA bypass circuit, we traced the fate of uniformly labeled (M6) ^13^C glucose in AMCMs exposed to ACLYi (BMS) or vehicle. As shown in Figure 2A, M6-glucose is converted by glycolysis into 2 M3-pyruvates, which can be converted to M3-lactate or decarboxylated in mitochondria to form M2-acetyl-CoA. M2-acetyl-CoA then enters the TCA cycle, generating M2-citrate and ultimately M2-malate. M2-citrate exiting mitochondria is converted by ACLY to M2-acetyl-CoA and unlabeled M0-OA. M0-OA is then converted to M0-malate and re-enters mitochondria to join the TCA cycle. Thus, if the ACLY-dependent bypass circuit is active, blocking ACLY should increase the level of M2-malate, and relative M2-malate/M2-citrate and M2-malate/M0-citrate ratios. Figure 2B shows that all 3 changes were observed after 1 hour ACLYi (1 μmol/L). Isotopologue fractions for other key metabolic intermediates are shown in Supplemental Figure S1A. Importantly, the M6-glucose input for both groups was the same (80% for each group, Supplemental Figure S1A). M3 pyruvate and M3 lactate both significantly increased following ACLYi indicating glycolysis was not inhibited and perhaps increased. Repeating the study with 10 μmol/L BMS yielded very similar changes (Supplemental Figure S1B) indicating that 1 μmol/L is sufficient.

### ACLYi induces LDH release from cardiomyocytes but not fibroblasts

AMCMs were incubated with BMS (1-50 μmol/L) for 1 or 4 hours to assess its impact on any cytotoxicity. Studies in hepatic, cancer, and vascular smooth muscle cells reported no toxicity even at the highest dose.^6^^,^^10^^,^^20^ In cardiomyocytes, however, we observed dose-dependent LDH release, a marker of cytotoxicity (Figure 2C), and this was not BMS specific because similar changes were obtained with other selective inhibitors (Supplemental Figure S2A). Importantly, at 1 μmol/L BMS, LDH release was not significant nor was there increased cell death detected by EthD-1 (Supplemental Figure S2B). This lower nontoxic dose was used in subsequent AMCM studies. NRCMs also displayed some cytotoxicity but only at the highest doses (Figure 2D). In contrast to myocytes, primary cardiac fibroblasts that also express ACLY^9^ exhibited no cytotoxicity at these BMS doses (Figure 2E). This pattern of LDH response to BMS was observed after 24 hours, with slightly higher levels in AMCMs (Supplemental Figure S2C).

### Effect of subtoxic ACLYi on rest and isoproterenol stimulated cardiomyocyte function

AMCMs were pre-exposed to 1 μmol/L ACLYi or vehicle for 1 hour, then electrically stimulated at 1 Hz and sarcomere shortening and calcium transients determined at rest and upon beta-receptor stimulation (isoproterenol, 10 μmol/L). Figure 2F shows example time-tracings for sarcomere shortening and Ca^2+^-transients and Figure 2G the summary data. Sarcomere shortening and calcium transients were similar with or without BMS, both at baseline and upon isoproterenol stimulation. Thus, at a nontoxic BMS dose still sufficient to impact citrate/malate metabolism, rest and stimulated myocyte function and calcium cycling were unaltered.

### ACLY controls NAD^+^/NADH balance in cardiomyocytes

**Figure 3:**
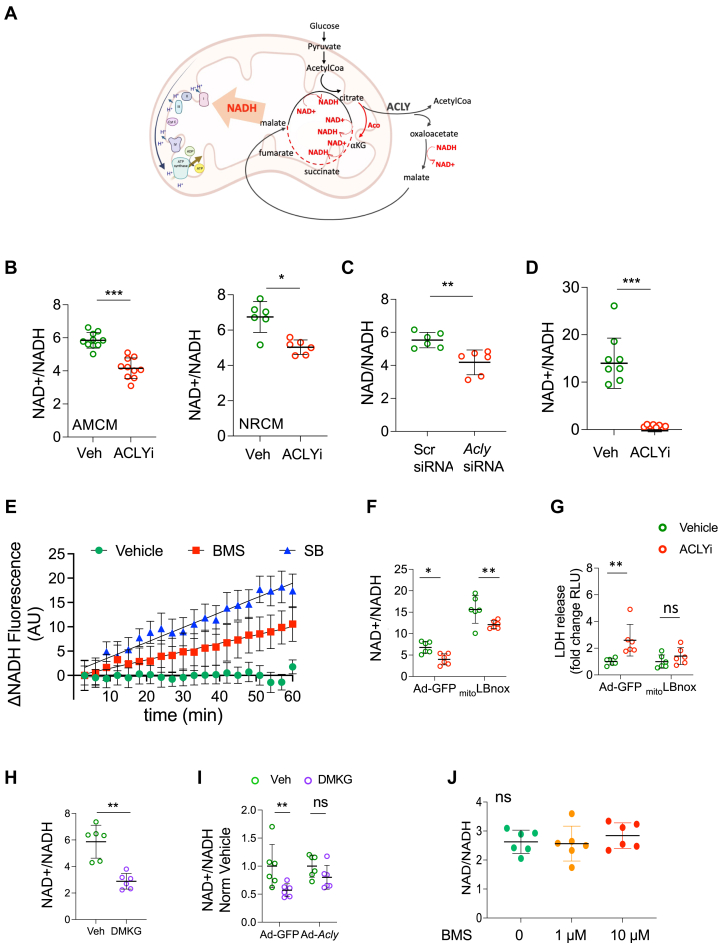
ACLY Disruption in Cardiomyocytes Reduces NAD^+^/NADH Ratio Associated With Cardiotoxicity, Which Is Alleviated by NAD+ Augmentation (A) Schematic of TCA cycle showing how ACLY inhibition would block cytosolic NADH consumption and enhance TCA cycle generation. (B) NAD^+^/NADH ratio in adult mouse cardiomyocytes (AMCM) and neonatal rat cardiomyocytes (NRCM) after 1 hour ACLYi (1 μmol/L, 25 μmol/L, respectively). Mann-Whitney *U* test, n = 6-10/group. (C) Same ratio measured in NRCM transfected by *Acly*-siRNA or scrambled siRNA, data measured after 24 hours of silencing. Mann-Whitney *U* test, n = 6/group. D- NAD^+^/NADH in isolated mitochondria from NRCM study in panel B. Mann-Whitney *U* test, n = 8/group. (E) Delta NADH (% reduced) autofluorescence measured in AMCM exposed to vehicle, BMS, or SB ACLY inhibitor (both at 0.25 μmol/L). Data shown are mean and SEM. Linear regression analysis with 2-way ANOVA, n = 10/group. (F) Total cellular NAD^+^/NADH and (G) LDH release in NRCM transduced with _mito_LBnox or control vector and treated with 25 μmol/L ACLYi vs vehicle. Two-way ANOVA with Sidak’s multiple comparison test. n = 6/condition. H-NAD^+^/NADH in NRCM exposed to dimethyl-α-ketoglutarate (DMKG) or vehicle. Mann-Whitney *U* test, n = 6/condition. I- NAD^+^/NADH in NRCMs transduced with AdV expressing green fluorescent protein (*Gfp)* or *Acly ±* DMKG. Data normalized to control in each group. Data shown are mean ± SD. Two-way ANOVA, n = 6/group. (J) NAD^+^/NADH ratio measured in primary adult cardiac fibroblasts. Mann-Whitney test, n = 6/group. ∗*P <* 0.05, ∗∗*P <* 0.01, and ∗∗∗*P <* 0.001. Except for panel E, all individual data are displayed in the figures along with mean ± SD.

The TCA cycle normally generates 3 NADH, but this declines to 1 while another is consumed in the cytosol with an active ACLY-dependent TCA bypass (Figure 3A). Thus, ACLYi should increase NADH and potentially lower NAD^+^/NADH ratio. Exposure to 1 hour ACLYi in both AMCMs and NRCMs consistently reduced NAD^+^/NADH ratio (Figure 3B) and was replicated by gene suppression of *Acly* by silencing RNA (siRNA) (Figure 3C, Supplemental Figure S3A). Rapidly isolated mitochondria from NRCMs pre-treated with 1 hour ACLYi had a very reduced NAD^+^/NADH ratio (Figure 3D) suggesting a rise in mitochondrial NADH. This was directly tested by real-time NADH auto-fluorescence in AMCMs (Figure 3E). NADH fluorescence signal was stable over an hour observation with vehicle, but rose significantly and linearly with time upon addition of either ACLY inhibitor BMS or SB.

To test whether ACLYi-induced cytotoxicity is coupled to a reduced NAD^+^/NADH ratio, NRCMs were infected with adenovirus expressing the mitochondrially targeted LB-nox enzyme (_mito_LBnox) that generates NAD^+^ and H_2_O from NADH.^21^ This raised resting NAD^+^/NADH with or without ACLYi (Figure 3F) but eliminated LDH release upon ACLYi seen in control subjects (Figure 3G). Using an opposite approach, myocytes were exposed to a membrane permeant form of the TCA cycle intermediate (di-methyl alpha-ketoglutarate) to stimulate this cycle and lower NAD+/NADH ratio (Figure 3H). Cells were also pretransfected with *Acly* or control (GFP). The fall in NAD+/NADH was observed in control subjects but prevented by *Acly* overexpression (Figure 3I). Last, because cardiac fibroblasts did not release LDH despite BMS exposure, we assayed NAD^+^/NADH ratio in these cells and found it unaltered vs control subjects (Figure 3J). Together, these data couple ACLY inhibition with a fall in NAD^+^/NADH caused by mitochondrial NADH increase, which is associated with myocyte cytotoxicity, and prevented by restoring NAD^+^/NADH balance.

### Influence of ACLYi on myocyte oxidant stress and mitochondrial respiration

**Figure 4:**
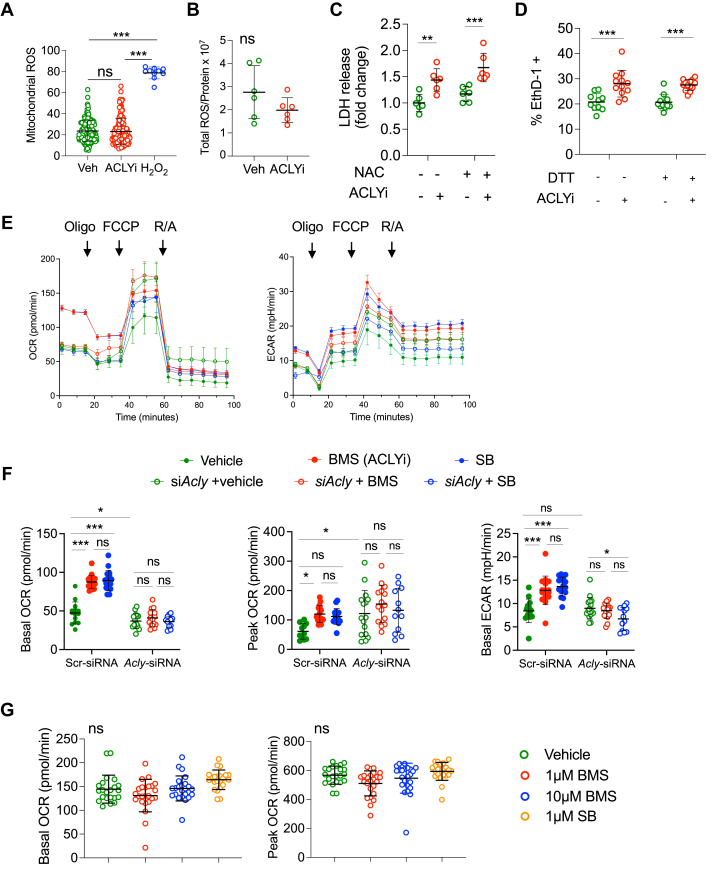
ACLYi Does Not Increase Myocyte ROS and Augments Mitochondrial Respiration A- Mitochondrial ROS assessed by mitoSox in NRCM after 1 hour of incubation with 25μM ACLYi, vehicle, or hydrogen peroxide (H_2_O_2_) as a control. Welch ANOVA, Dunnett’s multiple comparisons test. n = 160 cells per control and ACLYi, n = 10 cells for H_2_O_2_. B-Total cellular ROS by EPR normalized to total protein in NRCM ± 25 μM ACLYi for 1 hour. Mann-Whitney test, n = 6/group. C- LDH release (normalized to control) from NRCM exposed to 25 μM ACLYi vs vehicle x 1hr ± n-acetylcysteine (NAC). Two-Way ANOVA, Dunnett’s test, n = 6/group. D- Cell death detected by Ethd-1 staining in AMCM exposed to 1 μM BMS with or without anti-oxidant DTT. The increase with BMS is unaltered by DTT (*P <* 0.001 for control; *P* < 0.001 for DTT; *P =* 0.90 for interaction). Two-way ANOVA with Sidak’s multiple comparisons test, n = 10-13/group. E- Time plots of NRCM oxygen consumption rate (OCR) and extracellular acidification rate (ECAR) in cells pre-treated with *Acly*-siRNA or *scr*-siRNA (control) and then exposed to 1 hour BMS or SB, or vehicle. Data shown are mean ± SEM. n = 14-16/group. Oligo; oligomycin, FCCP; Carbonyl cyanide-p-trifluoromethoxyphenylhydrazone, R/A; rotenone + antimycin. F- dot plots of NRCM oxygen consumption rate (OCR) and extracellular acidification rate (ECAR) in cells pre-treated with *Acly*-siRNA or *scr*-siRNA (control) and then exposed to 1 hour BMS or SB, or vehicle. ANOVA with Sidak’s multiple comparisons test. n = 14-16/group G- Dot plots for isolated mitochondrial basal OCR and peak OCR in the presence of vehicle, BMS at 2 doses, or SB. There was no impact of the ACLY inhibitors on the response, consistent with ACLY being a cytosolic protein and the inhibitor effect on OCR in whole cells being ACLY-dependent. One-way ANOVA with Dunnett’s multiple comparisons test. n = 22-24/group. ∗*P <* 0.05, ∗∗*P <* 0.01 and ∗∗∗*P <* 0.001. With the exception of Panel E, all other data are displayed with corresponding mean ± SD.

A relative excess of NADH over NAD^+^ may depress the electron transport chain in part by inducing oxidative stress via electron leak at complex I.^22^ To assess this, ROS were measured by several methods before and after 1 hour of 25 μmol/L BMS, a mildly cytotoxic dose. Mitochondrial ROS (mitoSOX) (Figure 4A, Supplemental Figure S4A), ROS measured by EPR (Figure 4B), and cytosolic H_2_O_2_ detected by CytoORBP (Supplemental Figure S4B) were assessed, and all were unchanged despite BMS exposure. Furthermore, adding the reducing agent N-acetylcysteine did not block LDH release from ACLYi in NRCMs (Figure 4C), nor did adding dithiothreitolor (another reducing agent) blunt cell death from BMS in paced adult cardiomyocytes (Figure 4D). Together, these data indicate that while reductive stress was induced by ACLYi, this did not appear to trigger oxidative stress.

Although an acute increase in mitochondrial NADH upon ACLYi could potentially cause electron leak and impair electron transport and oxidative respiration, the lack of ROS suggested this did not occur. To more directly test this, oxygen consumption (OCR) and extracellular acidification (ECAR) rates were measured in NRCMs pre-transfected with either *Acly-*siRNA or scrambled control, and then exposed to 1hr ACLYi (BMS or SB). Both ACLY inhibitors induced a similar significant rise in basal, peak OCR, and ATP-coupled respiration with somewhat smaller rises in ECAR (Figures 4E and 4F, Supplemental Figure S3B). Cells with *Acly* gene knockdown also had higher peak OCR, and importantly, did not exhibit changes in OCR, ECAR, or ATP-coupled respiration with either ACLY inhibitor, supporting selectivity of these drugs for ACLY.

Because ACLY is cytosolic, isolated mitochondria should not exhibit changes in respiration from ACLYi so long as the inhibitors are specific to ACLY. This was tested (Figure 4G), and there was minimal effect of either BMS or SB on isolated mitochondrial respiration. The small ECAR rise with ACLYi is consistent with elevated lactate and pyruvate in the ^13^C-U6 glucose tracing study, but not so high as to suggest respiratory chain uncoupling. This was supported by assessment of mitochondrial membrane potential after ACLYi which was unaltered (Supplemental Figure S4C). Together, the data show that ACLY normally dampens oxidative respiration in resting cardiomyocytes, which rises upon ACLY inhibition.

### Genetic knock-down of ACLY depresses cardiac function in vivo

**Figure 5:**
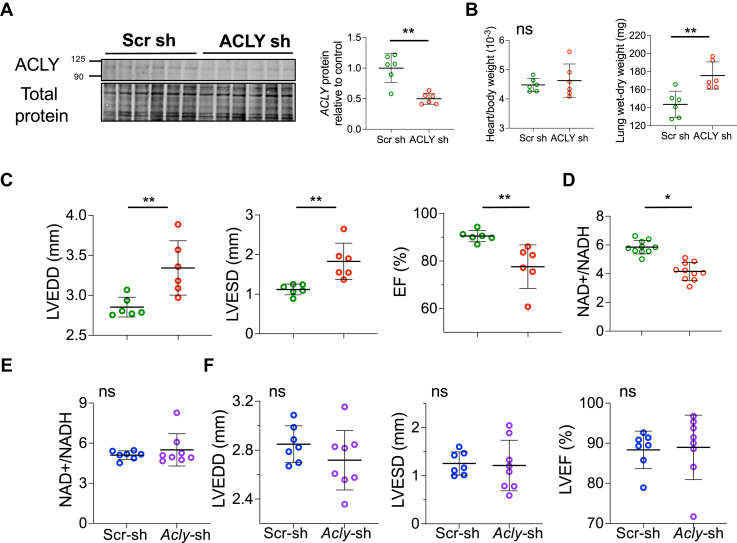
Effect of cardiac ACLY silencing by AAV9 shRNA in vivo (A) Myocardial ACLY protein expression from hearts transfected by AAV9-*Acly-*shRNA vs scramble control (Scr sh). (B) Heart weight/body weight, lung wet-dry weight. (C) Echo-cardiographic parameters: left ventricular end-diastolic diameters (LVEDD), left ventricular end-systolic diameter (LVESD), and left ventricular ejection fraction (LVEF) in same groups, Mann-Whitney *U* test, n = 6 mice/group. D- NAD^+^/NADH in myocardium of AAV9-*Acly*shRNA vs *scr*-shRNA treated mice. Mann-Whitney *U* test, n = 6/group. E- Same ratio measured in myocardium of both mouse groups after 6-week oral treatment with NMN (NAD supplementation). (F) Echocardiographic parameters in this study with NMN treatment in both groups, initiated 4 weeks after AAV9 inoculation. Mann-Whitney *U* test, n = 7-8 mice/group. Data in all panels are shown with mean ± SD. ∗*P <* 0.05, ∗∗*P <* 0.01, and ∗∗∗*P <* 0.001.

To test the effect of ACLY reduction on cardiac function in vivo, 2 models were studied. First, *Acly*-shRNA (or scrambled control) was delivered in an AAV9 vector and administered retro-orbitally to C57BL/6J mice. By 10 weeks postinjection, myocardial ACLY protein expression had declined by ∼50% (Figure 5A), similar to that found in human HF (c.f. Figure 1C). There was no change in heart/body weight, but lung wet-dry weight increased (Figure 5B) and end-diastolic and -systolic left ventricular (LV) volumes rose while ejection fraction (EF) significantly declined (Figure 5C). The NAD^+^/NADH ratio also fell in myocardium with *Acly* knockdown (Figure 5D). Because restoration of this ratio had blocked cytotoxicity from BMS in cardiomyocytes, we tested if restoring NAD^+^/NADH in vivo would counter these cardiac changes. AAV9-*Acly-*shRNA vs scrambled controls were fed an NAD+ precursor (NMN) in drinking water for 6 weeks. Myocardial NAD^+^/NADH ratio and ventricular morphology and function were no longer different between groups (Figures 5E and 5F) when animals received NMN supplementation in their drinking water.

**Figure 6:**
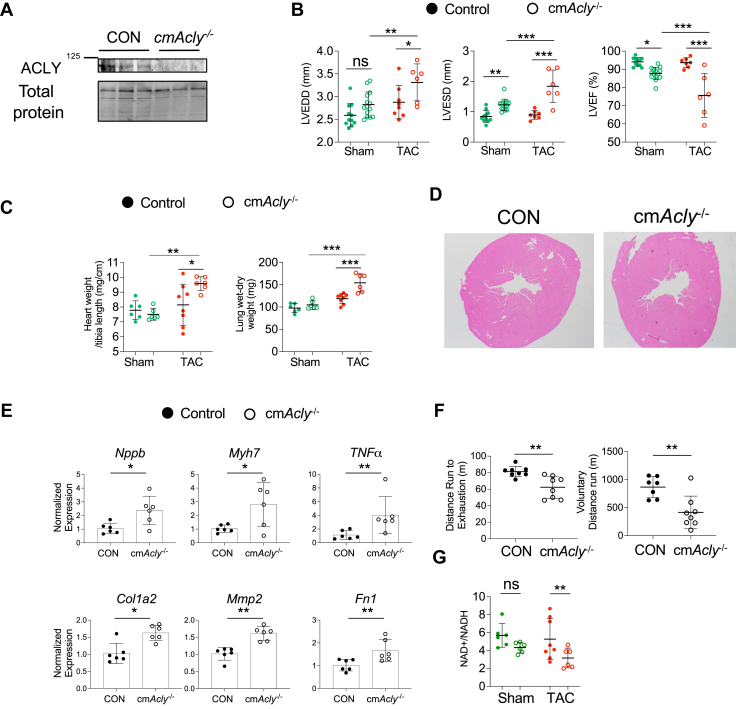
Influence of Cardiomyocyte-Selective Induced *Acly* KD In Vivo (A) Immunoblot of ACLY protein expression in cardiomyocytes from cm*Acly* KD mice and controls. B- LVEDD, LVESD, and LVEF at baseline and 8 weeks after mild transaortic constriction (TAC) in cm*Acly*KD and CON. Two-way ANOVA, Sidak’s multiple comparison test. n = 6-14 mice/group. (C) LV mass, lung wet-dry weight. Two-way ANOVA with Sidak’s multiple comparison test. n = 6-8 mice/group. (D) Examples of left ventricle cross-section for both groups at 2× magnification, displaying normal histology and negligible fibrosis. (E) Myocardial gene expression of stress and pro-fibrotic biomarkers—B-type natriuretic peptide (*Nppb*), b-myosin heavy chain (*Myh7*), tumor necrosis alpha (*Tnfa*), collagen type 1 (*Col1a2*), metalloprotease type 2 (*Mmp2*), and fibronectin (*Fn1*). Mann-Whitney *U* test. n = 6/group. (F) Distance run on exhaustion test and voluntary distance in cm*Acly* KD and controls (CON). Mann-Whitney *U* test, n = 7-8 mice/group. G) NAD+/NADH in myocardium from cm*Acly*KD vs control myocardium at baseline and 8 weeks after TAC. Two-way ANOVA, Sidak’s multiple comparison test; n = 6-14/group. Data in all panels are shown with mean ± SD. ∗*P <* 0.05, ∗∗*P <* 0.01, and ∗∗∗*P <* 0.001.

Because AAV9 can infect more than cardiomyocytes, we further tested the impact of reducing ACLY in cardiomyocytes only, using tamoxifen-inducible cm*Acly*^−/−^ mice. Cardiomyocyte ACLY protein expression was significantly reduced in cardiomyocytes (Figure 6A), and this was selective, being unchanged in total myocardium, liver, adipose tissue, and skeletal muscle (Supplemental Figure S5). Proteins in the electron transport chain (Supplemental Figure S6) and SREBP1 and CHREBP (Supplemental Figure S7) in isolated cardiomyocytes were unchanged between control subjects and cm*Acly*^*−/−*^. There were no changes in electron transport chain (ETC) proteins in skeletal muscle (Supplemental Figure S6). Resting heart function was mildly depressed in cm*Acly*^−/−^ vs control subjects, with no differences in LV mass or lung weight (green symbols in Figures 6B and 6C). There was negligible myocardial fibrosis in either group (Figure 6D, Supplemental Figure S8), but genes reflective of myocardial stress and fibrotic response were up-regulated in the cm*Acly*^−/−^ hearts (Figure 6E).

Despite the modestly reduced resting cardiac phenotype, cm*Acly*^*−/−*^ mice had considerably lower exertional capacity measured by treadmill test to exhaustion or voluntary exercise (Figure 2F), suggesting a potential impact on reserve. We tested for differences in acute cardiac reserve upon dobutamine stimulation by echo Doppler imaging in vivo (increasing resting cardiac output by 60%) and found responses in both groups essentially identical (Supplemental Figure S9A). For a more chronic stress, we applied mild pressure-overload induced by transaortic constriction (TAC), a hemodynamic stress that increases ACLY myocardial gene and protein expression (Supplemental Figures S9B and S9C), and measured cardiac adaptations by echocardiography. With chronic TAC, Cm-*Acly*^*−/−*^ mice exhibited greater chamber dilation and lower EF (Figure 6B), greater ventricular hypertrophy and wet lung weight (Figure 6C), and lower NAD^+^/NADH ratio (Figure 6G; red symbols in each graph show TAC data).

## Discussion

This study uncovers a role of ACLY in cardiomyocytes and the intact heart, namely supporting the NAD^+^/NADH balance to act as a brake on basal mitochondrial oxygen consumption and influence chronic cardiac stress adaptations. The lower NAD^+^/NADH ratio observed with pharmacological and genetically-induced ACLY suppression primarily was caused by a rise in mitochondrial NADH as demonstrated by a live autofluorescence assay. Although cytotoxicity from ACLYi was relatively modest, it occurred at lower doses of BMS compared with those found to be nontoxic in cancers and other proliferating cells.^10^^,^^20^ Even NRCMs that are proliferative were less sensitive to ACLYi than AMCMs Last, NAD+ restoration ameliorated cytotoxicity and improved in vivo function in hearts with *Acly* down-regulation, further supporting a key role of ACLY regulation of the NAD^+^/NADH ratio.

Redox dyshomeostasis in the heart has been traditionally viewed from the perspective of oxidative stress; however, various cardiac stressors can also result in excess reductive capacity, or reductive stress.^22^^,^^23^ This can occur from an overabundance of antioxidants such as reduced glutathione, hyperactivation of Nrf2-Keap1(NF-E2-related factor 2-Kelch-like-associated protein-1) signaling, or increased NADH over NAD^+^. NADH excess in particular is thought to induce cytotoxicity by impairing^24^ endoplasmic reticular function and protein folding that requires an oxidative micro-environment, and providing excess electrons to the electron transport chain that leads to electron leakage with superoxide formation at Complex I and depression of ATP synthesis.^22^ Although ACLYi lowered the NAD^+^/NADH ratio, it did not increase mitochondrial or cytosolic ROS or impair oxidative respiration, which was rather augmented, and its cytotoxic effects were unchanged by adding reducing agents. This suggests that reductive stress can have functional impacts on myocytes without requiring oxidative stress or breakdown of the ETC.

Our finding that OCR and ECAR rose upon ACLYi would support that the augmented NADH stimulated ETC-dependent oxidative metabolism. Preclinical models of hypertrophy and heart failure and human failing hearts often exhibit a decline in NAD^+^/NADH ratio, this often despite coexisting oxidative stress.^23^^,^^25^, ^26^, ^27^ Although the relative contributions of reduced NAD+ used in various enzymatic reactions and greater NADH likely vary, increasing this ratio with NAD+ enhancing therapy has been beneficial in multiple heart diseases.^26^^,^^28^, ^29^, ^30^ The decline in ACLY found in human HFrEF and HFpEF predicts a bias to NADH as a contributor to lower NAD^+^/NADH ratio, and in light of the current data, might be viewed as an adaptive response to enhance mitochondrial respiration in conditions where TCA-NADH generation is compromised.^31^ Alternatively, because NADH also potently inhibits citrate synthase, the rate-limiting step in the TCA cycle, and isocitrate dehydrogenase, depressed ACLY and consequent lowering of NAD^+^/NADH might be interpreted by the cell as fuel excess and so depress NADH-generating pathways. This could reduce function over time and blunt stress adaptations. Additional studies are needed to fully identify these mechanisms, but the current findings support the perspective of ACLY serving as an important mitochondrial redox-regulator in the heart.

There have only been a few studies using direct ACLY inhibitors in preclinical models in vivo, and BMS itself has been used at doses spanning a broad range (5-100 mg/kg/d).^6^^,^^7^^,^^20^^,^^32^ None reported any obvious cardiotoxicity—eg, animals developing a heart failure or some other cardiotoxic phenotype. In 2024, Grobs et al^6^ studied a model of pulmonary hypertension in rats and found no impact of 2 weeks of BMS at the low-end dose (5 mg/kg/d) on LV function. They also induced right ventricular hypertrophy by pulmonary artery banding, and found no cardiotoxic functional effects. This is not that different from our genetic model results in that the resting phenotype was mild, although our LV banding study did augment changes, and this in a model where only cardiomyocytes had reduced ACLY levels.

### Study limitations

While the results link reduced NAD^+^/NADH from ACLYi to cytotoxicity and in vivo functional effects, the precise mechanism by which this ratio is altered remains uncertain. Beyond the impact of NADH elevation, changes in NAD^+^ could influence a variety of enzymes for which this serves as a cofactor, such as sirtuins.^33^ Although such interactions could not explain changes we observed in 1 hour, chronic responses might involve these pathways as well. This may be quite difficult to determine, because there are many competing pathways and mechanisms by which NAD^+^/NADH is regulated, and even with our labeling studies, changes observed with ACLYi were difficult to link to a definitive cause. Finding an appropriate animal model that mimics ACLY down-regulation observed in human HF has also been difficult, as our initial survey of pressure-overload, diet-induced obesity, and an HFpEF model (L-NAME + high fat diet) showed that none exhibit such behavior.

## Conclusions

We identify a role of ACLY in myocytes and intact hearts to regulate the critical balance of NAD^+^/NADH. This appears to provide a regulatory release valve limiting excess mitochondrial NADH generation and impacting net mitochondrial respiration. Reduced ACLY activity has modest acute cytotoxic effects if applied directly to cardiomyocytes, these cells being more sensitive to ACLY inhibition than what is reported from proliferative cells such as cancer, vascular smooth muscle cells, stem cells, or myofibroblasts. Reduced ACLY expression in human HF may contribute to altered mitochondrial respiratory function and NAD^+^/NADH imbalance, and our findings add another molecular target for NAD+ supplementation therapy to treat HF conditions.

Perspectives **COMPETENCY IN MEDICAL KNOWLEDGE:** HF has been traditionally viewed and treated from the perspectives of hemodynamic, neurohormonal, and structural abnormalities. However, underlying disease in metabolism, the catabolism of various substrates, oxidative respiration, and control over critical substrates required for these processes also play a central role. The pandemic of obesity and cardiometabolic syndrome has made this challenging situation worse. As new therapies are developed for heart failure engaging metabolic pathways, knowledge of metabolic health and defects in disease has become more important. This study provides new insight into a mechanism by which the heart controls the balance of NAD+/NADH, a core metabolic regulator, associated with the enzyme ACLY.

**TRANSLATIONAL OUTLOOK:** One ACLY inhibitor (bempedoic acid) is currently approved to treat lipidemia in statin-intolerant patients, although the drug requires activation in the liver and so does not impact ACLY in other organs. Other direct inhibitors are in development for cancer, and there is interest for pulmonary hypertension and hepatic steatosis. Our finding that ACLY is expressed at lower levels in heart failure syndromes, and that reducing ACLY expression or its activity alters myocyte metabolism and energy balance with negative impact on rest and reserve function suggests care be applied as such therapeutics are developed.

## Funding Support and Author Disclosures

The work is supported by National Institutes of Health R35:135827 and R35:166565 (to Dr Kass), National Institutes of Health T32:HL007227 (to Dr Meddeb), the Belfer Endowment (to Dr Kass), American Heart Association Fellowship 23POST1026402 (to Dr Koleini), National Institutes of Health T32:HL007227 (to Dr Koleini), Amgen Inc (to Dr Sharma), National Heart, Lung, and Blood Institute 1K23HL166770-01 and 1L30HL138884 (to Dr Hahn), and NIAID: R01AI156274 (to Drs Pearce and Kass). All other authors have reported that they have no relationships relevant to the contents of this paper to disclose.
