# Supplementary material for: ATP-Citrate Lyase Supports Cardiac Function and NAD+/NADH Balance and Is Depressed in Human Failing Myocardium
Source: JACC Basic Transl Sci. 2025 Jun 10;10(7):101301. doi: 10.1016/j.jacbts.2025.04.015 (PMC12182301; doi:10.1016/j.jacbts.2025.04.015)
Supplement: Supplemental Material [file mmc1.docx]

**Supplemental Materials**

**Supplemental methods, tables, and figures with legends.**

**Supplemental Methods:**

**Reactive Oxygen Species Analysis**

*Electron Paramagnetic Resonance (EPR):* NRCM were treated with 25 μM of ACLYi or vehicle for 1 hour. After incubation, ROS was analyzed by electron paramagnetic resonance (EPR) spectroscopy, as previously described ^1^. The EPR signal intensities were normalized to the protein concentrations of the samples as determined by BCA protein assay kit (Thermofisher Scientific^TM^).

*Mitochondrial ROS:* NRCM were treated with 25 μM of ACLYi or vehicle for 1 hour. Cells treated with 10 mM hydrogen peroxide for 5 minutes were used as a positive control. The culture medium was subsequently replaced with PBS including 100 nM of MitoSOX^TM^ red reagent (Invitrogen #M36008). After 15 minutes of incubation with the MitoSOX^TM^ reagent, cells were imaged with a Revolve microscope (ECHO) using the rhodamine filter. The intensity of mitochondrial fluorescence was then measured using ImageJ software. The calculated fluorescence intensity after subtraction of background fluorescence for individual cells was used to compare mitochondrial ROS between groups.

*Cytosolic ROS:* NRCM were transduced with cytoORP1; an adenovirus construct expressing redox-sensitive GFP (roGFP) fused with the yeast peroxidase ORP1, localizing to the cytosol in live cells, as previously described ^2^, courtesy of Dr Brian O’Rourke’s laboratory. After 48 hours of transduction, at which time point a transduction efficiency of >90% was achieved, cells were treated with 25 μM of ACLYi or equal volumetric ratio of DMSO (vehicle) for 1 hour. The medium was subsequently changed to a modified Tyrode solution (10 mM glucose, 2 mM pyruvate, 130 mM NaCl, 5 mM KCl, 1 mmol MgCl_2_, 10 mM HEPES, 2 mM CaCl_2_ and 0.3mM ascorbic acid) and cells were imaged with an Andor XD Revolution spinning disk confocal microscope (Olympus) using 405- and 488-nm laser lines for excitation and 500- to 554nm bandpass filter for detection. Images were acquired every 2 seconds over 30 minutes, after which signals were calibrated using diamide to obtain the R_max_ for roGFP probe and dithiothreitol (DTT) to obtain the R_min_. ROS emission was then estimated by the 405/488 nm excitation ratio images that were created and analyzed using ImageJ software after normalization of the ratiometric signals to the R_min_ and R_max_.

**Assessment of mitochondrial NAD^+^/NADH ratio**

The NAD+/NADH ratio was measured using the NAD+/NADH-Glo^TM^ assay (Promega) according to the manufacturer’s instructions. Briefly, cells were washed with PBS and lysed in base solution containing 1% dodecyltrimethylammonium bromide which preserves the stability of the nucleotides. Taking advantage of the differential stability of the oxidized (NAD+ stable with heating in acidic PH) and the reduced form (NADH stable with heating in basic PH), the cells were then split into separate wells for acid and base treatments and heated at 60°C for 15 minutes. The acidic/basic solutions were then neutralized, and an equal volume of reconstituted NAD+/NADH Glo^TM^ detection reagent was added to each well. Reactions were incubated at room temperature for 60 minutes and luminescence was read using a Glomax microplate reader.

To measure mitochondrial NAD^+^/NADH balance, NRCM were transduced with a mitochondrial HA tag^3^ packaged into an adenovirus vector. Forty-eight hours later, cells were treated with 25 μM of ACLYi or equivalent volume of vehicle for 1 hour. One hour after treatment, cells were washed with PBS, lifted in fresh PBS with a cell lifter (Corning #3008) and mitochondria were isolated using Pierce^TM^ anti-HA magnetic beads (Thermo Scientific #88836) according to the manufacturer’s recommendation in the cold room. Isolated mitochondria were subsequently lysed and NAD^+^/NADH ratio immediately measured using the NAD^+^/NADH-Glo^TM^ assay as described above.

**Mitochondrial Isolation and Oxygen Consumption Analysis**

Briefly, 3 adult mouse hearts were harvested, and immediately immersed in 10 ml of cold isolation solution (IS) consisting of 0.7M mannitol, 0.21M sucrose, 0.1M EGTA and 0.5% BSA. The hearts were then washed and sliced in the IS, after which the IS was discarded and 1 ml of fresh IS supplemented with 0.2 mg of protease was added. The heart fragments were subsequently homogenized 2-3 times using a glass homogenizer (on ice) until the tissue was fully disrupted. The suspension was subjected to differential centrifugation to purify mitochondria. Purified mitochondria were resuspended in B buffer (KH_2_PO_4_ 2mM, KCL 137mM, MgCl_2_ 2.5mM, EGTA 0.5mM, PH=7.2) and mitochondrial protein concentration was measured using the Pierce BCA protein assay kit (Thermofisher). Then, 500μg of mitochondria was transferred to a tube, and the volumes were raised to 2.5ml using B buffer, pyruvate and malate at final concentrations of 10 and 2mM, respectively. Mitochondria was plated into wells of a polyethylenimine coated seahorse plate (25μl per well) and spun at 3000g for 15 minutes at 4C. 155μl of warm B buffer (supplemented with pyruvate and malate and vehicle or Acly inhibitors) were added to the wells. After 30 minutes of incubation in a non-CO2 incubator (37C), OCR was measured using a XF96 Seahorse analyzer. Adenosine diphosphate (ADP) 1mM, oligomycin 2.5 µg/mL, 2,4-dinitrophenol 5μM, and antimycin A 4μM, were serially injected and the changes in OCR were measured^4^.

**Mitochondrial membrane Potential**

Membrane potential in NRCM treated with 25 μM ACLYi or vehicle (DMSO) for 1 hr was measured by 20 nM tetramethylrhodamine, methyl ester (TMRM; Invitrogen #I34361) incubated for 30 minutes per manufacturer’s protocol. Fluorescence intensity was quantified by Image J (Fiji) and normalized to background.

**Real-time mitochondrial NADH autofluorescence assay**

Isolated adult cardiomyocytes were loaded into a heated chamber (37°C) on the stage of a fluorescence microscope and superfused with Tyrode´s solution containing (in mmol/L): NaCl 130, KCl 5, MgCl2 1, Na-HEPES 10, CaCl2 1.5, glucose 10; pH 7.4. Cells were then treated with 0.25uM BMS or SB for 1 hour and NADH autofluorescence imaged every 3 min with excitation at 360nm and emission at 450nm. NADH level was expressed as percent reduction of the NADH/NAD+ pool, calibrated by adding the cytochrome oxidase inhibitor 4 mM NaCN (100%) and the mitochondrial uncoupler 5 μM FCCP (0 %) at the end of each recording.

**Manipulation of NAD+/NADH balance using α-ketoglutarate in NRVMs:**

NRCM were treated with 2.5 mM dimethyl-α-ketoglutarate (DMKG; Cayman chemical #28394) pre-buffered to target pH of 7.4±0.2, or equivalent volume of normal saline (vehicle).

ACLY overexpression employed a custom adenovirus expressing human ACLY or a control GFP adenovirus vector. (supplemental methods).

**Mouse echocardiography and exercise studies**

Transthoracic echocardiography was obtained with a VisualSonics Vevo 2100 system with a 18-38mHz transducer (Fuji Film Inc, Toronto, Canada) in the JHU Small Animal Cardiovascular Phenotyping and Model Core, by an operator blinded to the study conditions (details in *Supplemental Methods).*

For exercise to exhaustion testing, mice were trained and acclimatized to treadmill exercise over 3 days. On the test day, mice first ran uphill (15°) for 5 min at 5.6 m/min with subsequent speeds of 14, 16, 20, 22, and 24 m/min increased every 2 minutes until exhaustion. Total run time and distance were calculated based on treadmill speed and time run at each stage.

Voluntary exercise was measured in mice using Wireless Running Mousercise™ (Tactile Robotics, Winnipeg, Canada). Mice were single-caged in rat cages containing a free rotating slanted disk placed within the cage for 24 hours. Mice had free access to food and water. After 24 hours, the number of revelations and the time mice were active were determined.

**RNA extraction and semiquantitative real time RT-PCR**

RNA extraction was performed using TRIzol^®^ reagent (ThermoFisher Scientific), according to the manufacturer’s recommendations. One hundred nanograms of RNA were reverse-transcribed using the Applied Biosystems^TM^ High-capacity cDNA Reverse Transcription Kit (Applied Biosystems^TM^ #4368814). Semiquantitative reverse transcription PCR (qRT-PCR) was performed on a Quantstudio 5 instrument (ThermoFisher Scientific) using Applied Biosystems^TM^ TaqMan^TM^ Fast Advanced Master Mix (Applied Biosystems #4444963), with specific TaqMan primers (**Supplemental Table 2**). Expression levels of all transcripts studied were normalized to RPS18 housekeeping gene expression level, and the relative change in gene expression in each experimental condition was compared with those of controls using the 2^-ΔΔCt^.

***ACLY* silencing in NRCM**

Freshly isolated NRCM were transfected 24 hours after plating, with scrambled control siRNA (ON-TARGETplus Non-targeting Pool, Horizon Discovery #D-001810-10), versus *ACLY* siRNA (ON-TARGETplus ACLY siRNA, Horizon Discovery #L-089773-02), at a concentration of 50 nM and 100 nM, using the Lipofectamine RNAiMAX Reagent (ThermoFisher Scientific #13778100), according to the manufacturer’s recommendations. The 100 nM concentration was used in all further studies in this manuscript.

**Protein expression analysis**

Cells were scraped in SDS lysis buffer supplemented with 1:100 Halt^TM^ Protease and Phosphatase inhibitor cocktail (100X) (Thermo Scientific #78446). The lysates were sonicated, heated, and centrifuged (14,000g for 15 minutes) to remove debris. Protein concentration was quantified in the supernatants using the bicinchoninic acid assay (BCA). Following SDS-PAGE, proteins were transferred to nitrocellulose membranes. Membranes were blocked in Intercept® (TBS) Blocking Buffer (Li-COR) for 1 hour at room temperature to stop nonspecific binding. Membranes were incubated overnight at 4°C with the primary Anti-ACLY antibody (Abcam, #ab40793, clone EP704Y) diluted 1:1000 in Intercept® T20 (TBS) Antibody Diluent (Li-COR), washed in TBS-Tween (TBS-T) the following morning, followed by 1 hour incubation in secondary antibody at 1:5000 dilution at room temperature, followed by 1 hour of thorough washing in TBS-T and visualized by infrared imaging using Odyssey (Li-COR). Total protein concentration was determined using Revert^TM^ 520 Total Protein Stain kit (Li-COR) according to the manufacturer’s instructions. Primary antibodies are listed in Supplemental Table 3.

**Generation of adenovirus for ACLY overexpression:**

An adenovirus plasmid expressing human *ACLY* (transcript# NM_001096.3), under CMV promoter and fused to GFP in its C-terminus (VectorBuilder). Replication deficient adenovirus plasmids were linearized using PacI enzyme (NEB, Cat# R0547) as per manufacturer protocol. Human embryonic kidney 293T cells (ATCC, Cat # CRL-3216) were transfected by the linearized plasmids (10 mg per over 95% confluence T25 flasks) using Lipofectamine 3000 (Thermofisher, Cat# L3000001) as per manufacturer protocol. The next day, the media was renewed on the cells, and cells were cultured for 7 days. To acquire the virus, cells were collected and resuspended in 1 ml of PBS and subjected to 3 times of freeze/thaw cycles. Lysates were centrifuged at 2000g for 10 minutes, and supernatants were collected. The supernatants were put on >95% confluence HEK293T cells in a T75 flask and cultured for 7 days. Cells were collected, and an active virus was acquired. The virus titer was estimated by serial dilution as previously described ^5^. Cells were transduced with the virus at a multiplicity of infection (MOI) of 20.

**Echocardiography Analysis**

M-mode images (short axis, mid-ventricular level) were obtained in conscious mice, and included LV anterior and posterior wall thickness (LVAW,d; LVPW,d), LV diameter at end-diastole and end-systole (LVIDD, LVESD), LV fractional shortening (FS), LV ejection fraction (EF). Images were analyzed with VisualSonics software and average values from at least 5 heart beats per animal are reported.

**Human endomyocardial tissue**

Human endomyocardial biopsies from HFpEF patients and donor heart control tissue was provided from an existing biobank at Johns Hopkins University as previously described^6^. The donor control tissue is provided by a collaboration with the University of Pennsylvania and Gift of Life Foundation. All tissue was from the mid-septum accessed from the RV side, stored in -160^o^ C freezers, and obtained under IRB approved protocols at the respective institutions. Details and results from transcriptomic analysis of these cohorts have been reported ^6^. Clinical characteristics of the groups is provided in Supplemental Table 1. None of the patients were treated with an ACLY inhibitor. Immunoblots were generated de novo for this study and were from different patients of the same three groups previously reported^6^.

**REFERENCES:**

1. Keceli G, Gupta A, Sourdon J, Gabr R, Schar M, Dey S, Tocchetti CG, Stuber A, Agrimi J, Zhang Y, Leppo M, Steenbergen C, Lai S, Yanek LR, O'Rourke B, Gerstenblith G, Bottomley PA, Wang Y, Paolocci N and Weiss RG. Mitochondrial Creatine Kinase Attenuates Pathologic Remodeling in Heart Failure. *Circ Res*. 2022;130:741-759.

2. Dey S, Sidor A and O'Rourke B. Compartment-specific Control of Reactive Oxygen Species Scavenging by Antioxidant Pathway Enzymes. *J Biol Chem*. 2016;291:11185-97.

3. Chen WW, Freinkman E and Sabatini DM. Rapid immunopurification of mitochondria for metabolite profiling and absolute quantification of matrix metabolites. *Nat Protoc*. 2017;12:2215-2231.

4. Sakamuri S, Sperling JA, Sure VN, Dholakia MH, Peterson NR, Rutkai I, Mahalingam PS, Satou R and Katakam PVG. Measurement of respiratory function in isolated cardiac mitochondria using Seahorse XFe24 Analyzer: applications for aging research. *Geroscience*. 2018;40:347-356.

5. Lock M, Korn M, Wilson J, Sena-Esteves M and Gao G. Measuring the Infectious Titer of Recombinant Adenovirus Using Tissue Culture Infection Dose 50% (TCID(50)) End-Point Dilution and Quantitative Polymerase Chain Reaction (qPCR). *Cold Spring Harb Protoc*. 2019;2019.

6. Hahn VS, Knutsdottir H, Luo X, Bedi K, Margulies KB, Haldar SM, Stolina M, Yin J, Khakoo AY, Vaishnav J, Bader JS, Kass DA and Sharma K. Myocardial Gene Expression Signatures in Human Heart Failure With Preserved Ejection Fraction. *Circulation*. 2021;143:120-134.

| **Table 1. Clinical characteristics of Control, HFrEF, and HFpEF groups. Full set used for RNA analysis, and a random selected subset for protein expression.** | | | | |
| --- | --- | --- | --- | --- |
|  | **Control (24)** | **HFpEF (50)** | **HFrEF (30)** | **P value** |
| Age, years | 55±12 | 59.5±11.8^††^ | 50.1±13.9 | 0.007 |
| Female Sex, n (%) | 10 (41.7%) | 30 (60.0%) | 10 (33.3%) | 0.06 |
| Medications |  |  |  |  |
| ACEi or ARB, n (%) | 6 (25.0%) | 26 (52.0%) | 20 (66.7%) ** | 0.009 |
| Beta Blocker, n (%) | 6 (25.0%) | 22 (44.0%) ^†††^ | 28 (93.3%) *** | <0.001 |
| Loop Diuretic, n (%) | 0 (0%) | 39 (78.0%)***^,†^ | 30 (100.0%)*** | <0.001 |
| Past Medical History |  |  |  |  |
| Hypertension, n (%) | 11 (45.8%) | 40 (80.0%)*^,†^ | 30 (100.0%)*** | <0.001 |
| Diabetes, n (%) | 3 (12.5%) | 26 (52.0%)** | 9 (30.0%) | 0.003 |
| Coronary artery disease, n (%) | 1 (4.2%) | 4 (8.0%) | 5 (16.7%) | 0.13 |
| BMI, kg/m^2^ | 27.4 ±7.6 | 44.1±9.4***^, †††^ | 26.7±4.8 | <0.001 |
| LVEF, % | 63.1±7.4 | 62.9±6.3^†††^ | 18±7*** | <0.001 |
| LV mass/height^1.7^, g/m^1.7^ | 125±35 | 99±34***^, †††^ | 166.6±43 | <0.001 |
| Data are n (%) or median (25th-75th percentile). Fisher's exact test used for categorical variables with additional Bonferroni correction for multiple comparisons. 1-Way ANOVA with Tukey’s multiple comparisons test was used for continuous variables. *p<0.05 vs Control, **p≤0.01 vs Control, ***p≤0.001 vs Control. †p<0.05 vs HFrEF, ††p≤0.01 vs HFrEF, †††p≤0.001 vs HFrEF. ACEi, angiotensin converting enzyme inhibitor; ARB, angiotensin II receptor blocker; BMI, body mass index; LVEF, left ventricular ejection fraction; LV, left ventricle; Sex-adjusted LV mass/height^1.7^ was calculated by multiplying by a constant of 1.28 for women. | | | | |

| **Table 2. Reference of TaqMan probes used for gene expression analyses** | | | |
| --- | --- | --- | --- |
| **Target** | **Species** | **Supplier** | **Reference** |
| RPS18 | mouse | Thermo Fisher Scientific | Mm02601777 |
| ACLY | mouse | Thermo Fisher Scientific | Mm01302282 |
| BNP | mouse | Thermo Fisher Scientific | Mm01255770 |
| MYH7 | mouse | Thermo Fisher Scientific | Mm00600555 |
| TNF-α | mouse | Thermo Fisher Scientific | Mm00443258 |
| Ctgf | mouse | Thermo Fisher Scientific | Mm01192933 |
| Col1a2 | mouse | Thermo Fisher Scientific | Mm01309565 |
| MMP2 | mouse | Thermo Fisher Scientific | Mm00439398 |
| Fibronectin | mouse | Thermo Fisher Scientific | Mm01256744 |

| **Table 3. Supplier and reference numbers for antibodies used for immunoblotting assays** | | | | |
| --- | --- | --- | --- | --- |
| **Target** | **Reactivity** | **Supplier** | **Reference** | **Clone** |
| ACLY | human, mouse, rat | Abcam | ab40793 | EP704Y |
| CHREBP | human, mouse, rat | Cell signaling | 58069S | NA |
| SREBP1 | human, mouse, rat, pig, monckey, chicken, bovine, goat | Proteintech | 14088-1-AP | AB_2255217 |
| Mitochondrial ETC complexes | human, mouse, rat, sheep | Thermofisher Scientific | 45-8099 | cocktail, AB_2533835 |
| Pan-acetylation | human, mouse, rat, drosophila | Proteintech | 66289-1-Ig | 1D5F2 |

**FIGURE S1: A:** Isotopologue distribution in adult mouse cardiomyocytes (AMCM) after 1 hour of uniformly-labeled C13 glucose tracing with vehicle versus 1μM ACLYi treatment. 2-way ANOVA with Sidak’s multiple comparisons test. N=6 wells/condition. Only P values <0.05 are shown. **B:** Citrate isotopologue distribution, M2 malate/M2 citrate, and M2 malate/M0 malate ratios for cardiomyocytes treated with 10μM ACLYi versus vehicle for comparison with ANOVA with Sidak’s multiple comparisons test was used for isotopologue comparison. Mann-Whitney test used for ratios. N=5-6 wells/condition. All data shown are mean±SD. *p<0.05, **p<0.01 and ***p<0.001.

**FIGURE S2: A -** Lactate dehydrogenase (LDH) releases from adult mouse cardiomyocytes after 1 hour, 4 hours and 24 hours of exposure to vehicle versus the ACLY inhibitors SB-204990 (SB), NDI-091143 (NDI) and 10,11-dehydrocurvalarin (DH) (BA), used at 1μM and 10μM concentrations. The hepatocyte-specific ACLY inhibitor pro-drug bempedoic acid was used as an additional control at a 50μM concentration. Statistical analysis using ANOVA with Dunnett’s multiple comparison test. Only significant p values <0.05 are shown. N= 6-24 wells/condition. **B-** Percentage of adult mouse cardiomyocytes with ethidium homodimer-1 (EthD-1) positivity (reflecting cellular death) after 1 hour of exposure to vehicle versus 1μM of the ACLY inhibitor BMS 303141. Mann Whitney test. N= 4-7 wells/condition. **C-** Lactate dehydrogenase (LDH) releases from adult mouse cardiomyocytes (AMCM), neonatal rat ventricular myocytes (NRCM) and cardiac fibroblasts (cFb) after 24 hours of incubation with the ACLY inhibitor BMS 303141 at varying concentrations. ANOVA with Dunnett’s multiple comparison test. N= 5-6 wells/condition. All data shown are mean±SD. *p<0.05, **p<0.01 and ***p<0.001.

**FIGURE S3: A-**Immunoblot of ACLY expression in NRCM transfected with either control scrambled siRNA (Scr siRNA) or ACLY siRNA at 50 nM and 100 nM concentration. The 100 nM concentration was used in subsequent studies in the study. **B**- ATP-coupled respiration for NRCM study shown in Figure 4E, 4F. Data are normalized to vehicle control for each group, analysis by two-way ANOVA, Šídák's multiple comparisons test p-values shown. N=14-16/group. Data shown are mean±SD. *p<0.05, **p<0.01 and ***p<0.001.

**FIGURE S4: A** Representative confocal microscopy images of neonatal rat ventricular myocytes (NRCM) stained with mitoSox^TM^ after 1 hour of incubation with vehicle, 25 mM ACLYi or hydrogen peroxide (H_2_O_2_, positive control). **B-** Cytosolic reactive oxygen species assessed using a cytosolic ROS sensor (cytoORBP) in NRCM after 1 hour of treatment with vehicle versus 25 mM ACLYi. Comparison using Mann-Whitney test. N=10-13 fields per condition.  **C-** Representative confocal fluorescence microscopy images of neonatal rat cardiomyocytes (NRCM) stained with Tetramethylrhodamine, methylester, Perchlorate (TMRM) after 1 hour of treatment with 25 μM with ACLYi. TMRM intensities normalized to background were compared between vehicle-treated and ACLYi-treated cells. Statistical analysis using t-test. N= 50-60 cells/condition. All data shown are mean±SD. *p<0.05, **p<0.01 and ***p<0.001.

**FIGURE S5:** Immunoblots for ACLY protein normalized to total protein and then to the mean level in the control (Con) group for total myocardium (heart), adipose tissue, liver, and skeletal muscle for Con versus cardiomyocyte-specific *ACLY* knockout mice ( cm*ACLY* -/-). P values using Mann-Whitney test, N= 6 animals/group. All data shown are mean±SD. *p<0.05, **p<0.01 and ***p<0.001.

**FIGURE S6:** **A-**Immunoblot for mitochondrial electron transport chain (ETC) complex proteins (C-V-ATP5A, C-III-UQCRC2, C-IV-MTCO1, C-II-SDHB and C-I-NDUFB8) in isolated cardiomyocytes from control (Con) and cardiomyocyte-specific *ACLY* knockout mice ( cm*ACLY* -/-). Total protein is below . **B-** Summary data from both tissue sources for each ETC complex protein, with data normalized to total protein and then to mean of the Control group. P values are using Mann-Whitney test, N=6 animals/group. All data shown are mean±SD. *p<0.05, **p<0.01 and ***p<0.001.

**FIGURE S7:** Immunoblot and summary densitometry results for Sterol regulatory element-binding protein 1 (SREBP-1), and carbohydrate response element-binding protein (CHREBP), each normalized to total protein and then to the mean of the control (Con) group. Same two mouse groups as in Figure S7; p values from Mann-Whitney test; N=5 animals/group. All data shown are mean±SD. *p<0.05, **p<0.01 and ***p<0.001.

**FIGURE S8:** Representative higher magnification (10x) images for hearts shown in Figure 6D (quadrant from left ventricular cross section) stained with hematoxylin and eosin-staining. **A-** control versus cardiomyocyte-specific *ACLY* knockout mice (cm*ACLY* -/-) and **B**- from scrambled (scr) shRNA versus *Acly*-shRNA -adeno-associated virus 9 (AAV9) transfected hearts in vivo. *p<0.05, **p<0.01 and ***p<0.001.

**Figure S9:** **A-** Change in heart rate (ΔHR), end-diastolic left ventricular volume (ΔED Volume), end-systolic left ventricular volume (ΔES Volume), cardiac output (ΔCO), left ventricular ejection fraction (ΔEF %) and isovolumic relaxation time (ΔIVRT) in control (Con) versus cardiomyocyte-specific *ACLY* knockout mice ( cm*ACLY*^-/-^) induced by i.p. dobutamine 75μg/30 g body weight **B-** Change in ACLY transcriptional level of wild-type C57bL6J mice after 8 weeks of transaortic constriction (TAC) versus sham. Mann-Whitney test. N= 7 mice/group. **C-** Western blot of ACLY and total protein in isolated cardiomyocytes of wild-type mice after 8 weeks of TAC versus sham. ACLY levels were normalized to total proteins. Mann-Whitney test. N=3 mice/group. All data shown are mean±SD. *p<0.05, **p<0.01 and ***p<0.001.
